# The effect of the lower instrumented vertebra (LIV) on pain and quality of life in patients surgically treated for an idiopathic scoliosis

**DOI:** 10.1186/1748-7161-7-S1-P16

**Published:** 2012-01-27

**Authors:** J Sanchez-Raya, J Bago

**Affiliations:** 1 Hospital Vall d’Hebron, Barcelona, Spain

## Purpose

The objectives of the study are: 1) to measure lumbar spine mobility and analyze the influence of the lower instrumented vertebra (LIV) in patients treated surgically for idiopathic scoliosis, and 2) to determine the correlation between the LIV, lumbar mobility, pain, and quality of life.

## Materials and methods

40 patients (36 women, 4 men) were included (mean age 27 years); average time since surgery was 135 months. The mean residual major curve Cobb angle was 35.4°. Lumbar mobility was measured during forward and lateral lumbar flexions using a dual digital inclinometer.

Patients fulfilled the SRS22 Questionnaire and the Quality of Life in Spine Deformities Profile to estimate the perceived sensation of rigidity. Additionally, they rated the pain in the low back area.

## Results

Lumbar mobility decreased in relation to the LIV. There was no correlation between lumbar range of motion and pain. The LIV do not correlated with the sensation of rigidity. The sensation of rigidity correlated both with SRS22 pain scale and the intensity of low back pain.

Health-related quality of life was moderately correlated with the LIV and lumbar mobility.

## Conclusions

The LIV has an influence on lumbar mobility. The intensity of low back pain is mild in these patients and is not related to the LIV nor to lumbar mobility. The subjective perception of rigidity does not correspond to the loss of lumbar mobility, but rather, is determined by the coexistence of pain. The LIV has a moderate effect on the global quality of life.

